# Supplementary material for: Measuring Stereotypes in Interprofessional Education: A Pilot High-Fidelity Simulation Study Among Postgraduate Nursing and Physician Students in a Spanish University
Source: Healthcare (Basel). 2024 Dec 5;12(23):2449. doi: 10.3390/healthcare12232449 (PMC11641475; doi:10.3390/healthcare12232449)
Supplement: Supplementary file 1 [file healthcare-12-02449-s001.zip › healthcare-3251208-supplementary.pdf]

Measuring Stereotypes in Interprofessional Education: A High-Fidelity Simulation Experience for Postgraduate Nursing and Physicians Students in Spain.

Supplementary Files.

Data Tables.

Table S1. Descriptive Statistics Items. All participants.

	Item1	Item2	Item3	Item4	Item5	Item6	Item7	Item8	Item9	Imte10	Item11	Item12	Item13
**Mean**	2.09	1,77	3,23	3,04	3,68	1,81	3,86	3,13	3,41	3,23	1,68	1,45	4,27
**Median**	2	2	4	3	4	2	4	3	3,5	3	1	1	4,5
**Mode**	2	1	4	2	5	1	4	4	2	3	1	1	5
**ST**	0.68	0,92	1,31	1,13	1,43	0,85	1,16	0,94	1,29	0,92	0,95	0,51	0,98
**Skewness**	-0,11	1,29	-0,46	0,12	-0,67	0,88	-1,49	0,084	-0,14	0,31	1,46	0,196	-1,9
**Kurtosis**	-0,65	1.34	-1,09	0,49	-0,99	0,42	1,89	-1,20	-1,32	-0,57	1,55	-2,16	4,82
**Min**	1	1	1	1	1	1	1	2	1	2	1	1	1
**Max**	3	4	5	5	5	4	5	5	5	5	4	4	5

Table S2. Descriptive Statistics Items. Nursing postgraduate student Group.

	Item1	Item2	Item3	Item4	Item5	Item6	Item7	Item8	Item9	Imte10	Item11	Item12	Item13
**Mean**	1,93	1,40	3,47	2,87	3,40	1,80	3,93	3,40	3,60	3,07	1,47	1,33	4,27
**Median**	2,00	1,00	4,00	3,00	4,00	2,00	4,00	4,00	4,00	3,00	1,00	1,00	4,00
**Mode**	2,00	1,00	4,00	2,00	4,00	1,00	4,00	4,00	5,00	3,00	1,00	1,00	5,00
**ST**	0,70	0,51	1,06	1,06	1,45	0,94	1,16	0,91	1,40	1,03	0,64	0,49	0,88
**Skewness**	-0,11	0,69	-0,20	-0,08	-0,11	1,07	-1,37	-0,19	-0,27	0,93	0,98	0,32	-1,24
**Kurtosis**	-0,67	-2,09	-1,14	-1,56	-1,19	0,50	1,95	-0,71	-1,26	-0,30	0,40	-1,62	1,82
**Min**	3	2	5	4	5	4	5	5	5	5	3	2	5
**Max**	1	1	2	1	1	1	1	2	1	2	1	1	2

Table S3. Descriptive Statistics Items. Physicians postgraduate student Group.

	Item1	Item2	Item3	Item4	Item5	Item6	Item7	Item8	Item9	Imte10	Item11	Item12	Item13
**Mean**	2,20	2,20	2,60	3,60	4,40	1,80	3,60	2,60	3,00	3,20	2,00	1,60	4,20
**Median**	2,00	2,00	3,00	3,00	5,00	2,00	4,00	3,00	3,00	3,00	2,00	2,00	4,00
**Mode**	2,00	2,00	1,00	3,00	5,00	1,00	4,00	3,00	4,00	3,00	1,00	2,00	5,00
**ST**	0,45	1,10	1,52	0,89	0,89	0,84	1,52	0,55	1,00	0,84	1,22	0,55	0,84
**Skewness**	0,00	1,36	-1,12	0,54	7,81	0,00	0,61	0,51	-0,40	-2,24	1,71	0,61	-1,26
**kurtosis**	5,00	2,92	-3,08	0,31	0,31	-0,61	3,72	-3,33	-3,00	-0,61	2,00	-3,33	-0,61
**Min**	3	4	4	5	5	3	5	3	4	4	4	2	5
**Max**	2	1	1	3	3	1	1	2	2	2	1	1	3

Table S4. Shapiro-Wilk normality analysis.

	Item1	Item2	Item3	Item4	Item5	Item6	Item7	Item8	Item9	Imte10	Item11	Item12	Item13
W-stat	0,784	0,697	0,880	0,895	0,829	0,824	0,755	0,863	0,888	0,871	0,711	0,633	0,784
p-value	0,000	0,000	0,015	0,028	0,002	0,002	0,000	0,007	0,020	0,010	0,000	0,000	0,000
alpha	0,05	0,05	0,05	0,05	0,05	0,05	0,05	0,05	0,05	0,05	0,05	0,05	0,05
normal	no	no	no	no	no	no	no	no	no	no	no	no	no

Table S5. Frequency responce distribution and percentages by item and group.

	**Nursing postgraduate student**					**Total**					**Physicians postgraduate student**				
	1++	2+	3	4-	5--	1	2	3	4	5	1	2	3	4	5
**Item 1**	4	**8**	4	0	0	4	**13**	5	0	0	0	**5**	1	0	0
	25	**50**	25			18,2	**59,1**	22,7				**83,3**	16,7		
**Item 2**	**9**	6	1	0	0	10	**10**	1	1	0	1	**4**	0	1	0
	**56,2**	37,5	6,25			45,5	**45,5**	4,5	4,5		16,6	**66,7**		16,6	
**Item 3**	0	4	2	**8**	2	2	4	3	**10**	3	2	0	1	**2**	1
		25	12,5	**50**	12,5	9.1	18,8	13,6	**45,5**	13,6	33,3		16.7	**33.3**	16,6
**Item 4**	1	**7**	2	6	0	1	**8**	5	7	1	0	1	**3**	1	1
	6,25	**43,7**	12,5	37,5		4.5	**36,5**	22,7	31,8	4,5		16,7	**50**	16,7	16.7
**Item 5**	2	4	1	**5**	4	2	4	2	6	**8**	0	0	1	1	**4**
	12,5	25	6,2	**31,2**	25	9	18,2	9	27,3	**36,4**			16,6	16,7	**66,6**
**Item 6**	**7**	6	2	1	0	**9**	8	4	1	0	**2**	2	2	0	0
	**43,7**	37,5	12,5	6,2		**40,1**	36,4	18,2	4,5		**33,3**	33,3	33,3		
**Item 7**	1	1	1	**8**	5	2	1	1	**12**	6	1	0	0	4	1
	6,2	6,2	6,2	**50**	31,2	9.1	4,5	4,5	**54,5**	27,2	16,2			66,6	16,2
**Item 8**	0	3	4	**8**	1	0	6	7	**8**	1	0	**3 50**	3	0	0
		18,7	25	**50**	16,2		27,3	31,8	**36,4**	4,5			50		
**Item 9**	1	3	3	3	**6**	1	5	5	5	**6**	0	**2**	2	2	0
	6,2	18,7	18,7	18,7	**37,5**	4.5	22,7	22,7	22,7	**27,3**		**33,3**	33,3	33,3	
**Item 10**	0	5	**7**	2	2	0	6	**9**	5	2	0	1	2	**3**	0
		31,2	**43,7**	12,5	12,5		27,2	**40,9**	22,7	9,1		16,6	33,3	**50**	
**Item 11**	**9**	6	1	0	0	**12**	8	1	1	0	**3**	2	0	1	0
	**56,2**	37,5	6,2			**54,5**	36,4	4,5	4,5		**50**	33,3		16,7	
**Item 12**	**10**	6	0	0	0	**12**	10	0	0	0	2	**4**	0	0	0
	**62,5**	37,5				**54,5**	45,4				33,3	**66,7**			
**Item 13**	0	1	1	7	**7**	1	1	2	9	**9**	1	0	1	2	**2**
		6,2	6,2	43,7	**43,7**	4.5	4,5	9,1	40,1	**40,1**	16,7		16,7	33,3	**33,3**
